# Editorial January 2016

**DOI:** 10.1098/rsos.150712

**Published:** 2016-01-20

**Authors:** Jeremy K. M. Sanders

*Royal Society Open Science* provides an exciting new avenue for researchers across all scientific disciplines to publish their research. It offers a range of innovative features backed by one of the world's most respected scientific institutions, so I was delighted to be invited to become the first Editor-in-Chief.

The key features that make *Open Science* different are:

— It covers the whole of science, from Pure Mathematics to Psychology, and it particularly welcomes work that cuts across conventional boundaries. The recent special call for papers on City Analytics is a good example, using the emerging tools of Big Data, complexity science and dynamical systems to bring about new understanding in areas such as crowd behaviour, urban planning and energy usage.
— Gold Open Access, currently with a full introductory waiver of the article processing charge: it is free to publish.
— The option of open peer review, with reviewers’ comments being published alongside the paper. Currently, approximately 60% of authors take up this option.
— Objective peer review, which means that all research deemed scientifically sound and useful to the community will be published. Priority and impact are not considerations.
— Open data principles, requiring all supporting data to be made available such that all results presented are fully reproducible. We work with several large data repositories to ensure that it is as easy as possible for authors to comply with this policy.
— Compatibility with prior deposit on a pre-print server. We have integrated our submission system with ArXiv so that authors can submit files to us just by entering their ArXiv ID number.
— Registered Reports, in which methods and proposed analyses are pre-registered and peer-reviewed prior to research being conducted. High-quality protocols are then provisionally accepted for publication before data collection commences. Once the study is completed, the author will finish the article including results and discussion sections. This will be appraised by the reviewers, and provided necessary conditions are met, will be published. We are the first broad scope journal to offer this type of article, broadening availability to disciplines other than psychology, neuroscience and economics. The format is open to attempts of replication as well as novel studies.
— Advanced analytics, allowing authors and readers to see how the publication has been received in social media and other non-conventional fora as well as citation performance.

In the 20 months since opening for submissions, *Open Science* has received 1050 original submissions and has accepted 332 papers for publication. The most-read articles have included such titles as

— An investigation of the false discovery rate and the misinterpretation of *p*-values [1];
— Omura's whales (*Balaenoptera omurai*) off northwest Madagascar: ecology, behaviour and conservation needs [2];
— The deep sea is a major sink for microplastic debris [3];
— The evolution of popular music: USA 1960–2010 [4]; and
— The advantage of short paper titles [5].

Open access is a way of publishing that has grown more quickly in biology than in many other areas, and the content of the papers published in the journal so far reflects that cultural difference. It has recently been agreed that the chemistry section of the journal will be published in collaboration with the Royal Society of Chemistry and so will benefit from their experience and reputation for managing and editing chemical science journals of the highest quality.

As it happens, I am a chemist. My research interests have spanned across many boundaries, and over the years I have published in journals covering Biochemistry, Pharmacology and Chemical Physics as well as in a broad range of core chemistry journals. In Cambridge, I have been Head of Physical Sciences and then Pro-Vice-Chancellor, roles that I enjoyed because of the broad intellectual sweep of my responsibilities. I have been Associate Editor of *New Journal of Chemistry* and Chair of the Board of *Chemical Society Reviews*, so I hope to bring some experience of running journals to this new role too. The journal has already begun to flourish through the terrific contributions of the Subject Editors and the publishing staff. I am greatly looking forward to working with them and of course with authors so that together we can develop *Royal Society Open Science* into an irresistible place for us all to publish our science.
